# Case Report and Literature Review of *Parvimonas micra*: Difficult-to-Treat Arthritis in Hiding

**DOI:** 10.3390/healthcare11212879

**Published:** 2023-11-01

**Authors:** Xabier Cenicacelaya Olabarrieta, Margarita Cabello Vallejo, José Luis Morell-Hita, Cristina Macía-Villa

**Affiliations:** 1 Department of Rheumatology, Ramón y Cajal University Hospital—IRYCIS, 28034 Madrid, Spain; xcenica@hotmail.com (X.C.O.); jlorozco13@hotmail.com (J.L.M.-H.); 2 Department of Microbiology, Ramón y Cajal University Hospital—IRYCIS, 28034 Madrid, Spain; margacabello96@gmail.com

**Keywords:** septic arthritis, arthritis, crystal arthropathies, chondrocalcinosis, neoplasms

## Abstract

Septic arthritis is a life-threatening rheumatological syndrome that is highly related to a patient’s immune status and comorbidities, and although the most common clinical presentation is rapid-onset monoarthritis, it can also appear as subacute or chronic joint swelling. In these cases, differential diagnosis is more challenging, but early diagnosis and treatment is no less urgent to ensure a good global prognosis and the best outcome of the affected joint. Anaerobic microorganisms, such as *Parvimonas micra*, are an uncommon cause of septic arthritis (less than 5% of cases) but may be the cause of subacute arthritis. Knowledge about *Parvimonas micra* is important, as it is difficult to culture in the laboratory and generates a synovial fluid with atypical characteristics for septic arthritis so that, if not suspected, its diagnosis can be easily overlooked and underdiagnosed. We present the case of a 76-year-old woman with subacute arthritis of the left knee, describe the difficult diagnosis and treatment of its unexpected cause (*Parvimonas micra*), and review previously described cases, identifying the possible common comorbidities that may help clinicians easily find and treat this cause of subacute septic arthritis.

## 1. Introduction

Septic arthritis is one of the classical rheumatological syndromes, with an annual incidence that varies from 1 to 35 cases per 100,000 individuals in different countries. Although any joint is vulnerable to infection, the knee is the most common site of septic arthritis (almost half of all cases), followed by the hip, shoulder, elbow, and ankle [1]. It usually originates from peri- or intra-articular inoculation, local trauma, postoperative surgery or a distant septic focus, and both the immune status of the patient and the virulence of the microorganism are the main factors involved in its pathogenesis [2]. In this sense, older age, autoimmune diseases, immunosuppressive therapy or conditions, crystal arthropathies, joint surgery, joint prosthesis, intraarticular procedures, local skin infections, etc., are described as the possible risk factors [3]. As septic arthritis is considered one of the main rheumatological emergencies due to its high morbidity and mortality, the quickest identification of the clinical picture and, if possible, its causative pathogen can be vital for the patient.

The most common clinical presentation is acute (rapid onset) arthritis with local erythema, increased temperature, swelling and pain of the affected joint with severe functional limitation, often affecting the general condition of the patient with high fever [1]. This clinical spectrum, coupled with high acute blood reactant factors, such as the erythrocyte sedimentation rate (ESR) and C-reactive protein (CRP), and a typical synovial fluid with a high percentage of leukocytes (predominantly neutrophils), low glucose and high protein levels, facilitate the differential diagnosis. In addition, the causal microbe is frequently found in blood and/or synovial fluid cultures.

However, there are other scenarios in which joint infection may occur atypically in the form of subacute or chronic arthritis. These subtypes constitute a diagnostic challenge for the rheumatologist, as they may initially be attributed to mechanical pathologies (i.e., osteoarthritis) or noninfectious inflammatory diseases (i.e., spondyloarthritis). This subacute and chronic course can be caused by anaerobic microorganisms, such as *Parvimonas micra*, a Gram-positive anaerobic coccus found mainly in the oral cavity, respiratory system, and gastrointestinal and female genitourinary tracts. It is rare to find this microorganism outside these locations; however, cases of bacteriemia, endocarditis, septic arthritis, osteomyelitis, and abscesses have been reported [4,5,6,7,8].

The prevalence of septic arthritis caused by *Parvimonas micra* is unknown. The low awareness of this microorganism as a possible cause of septic arthritis, together with the fact that it is technically difficult to isolate and the atypical characteristics of the synovial fluid [2], yield the “perfect storm” for it to be an underdiagnosed cause of septic arthritis. For these reasons, it is important to improve our knowledge about this bacterium as a possible cause of septic arthritis.

## 2. Detailed Case Description

**Figure 1:**
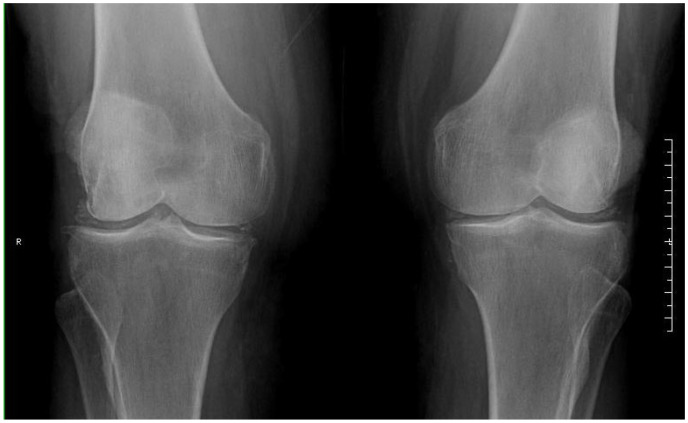
X-ray of the patient’s knees. Osteoarthritis (decreased femorotibial joint spaces and osteophytes) and radiographic chondrocalcinosis.

We present the case of a 76-year-old Caucasian woman with a personal history of hypertension, type 2 diabetes, dyslipidaemia and breast ductal carcinoma in remission after local surgery in 2013. She had been under follow-up in the rheumatology outpatient clinic since 2019 with a diagnosis of osteoarthritis in the left knee and radiographic chondrocalcinosis. She had no family history of chondrocalcinosis. She had never presented with joint inflammation beforehand and was treated with analgesics on demand with good control. In August 2021, the patient reported progressive mechanical pain in her left knee for two months. Physical examination revealed moderate swelling of the left knee. Local arthrocentesis was performed, with extraction of 45 cc of fluid and visualization of calcium pyrophosphate crystals on polarized light microscopy images and with the following laboratory characteristics: white blood cells (WBCs) 44,500/mL (95.5% neutrophils), Gram stain without microorganisms, and sterile cultures after 72 h. Local intra-articular corticosteroids were injected after checking the sample collection result. An X-ray of the left knee was performed (Figure 1), which showed the already-known signs of chondrocalcinosis and decreased medial femorotibial and femoropatellar joint spaces without erosions.

The patient returned three weeks later with no clinical changes, persistent pain, and swelling of the left knee. A second knee arthrocentesis was performed, with extraction of 30 cc of synovial fluid with the same polarized light microscopy findings as the previous arthrocentesis. The laboratory characteristics of this second synovial fluid were as follows: white blood cells (WBCs) 49,100/mL (94.7% neutrophils) and isolation of Gram-positive cocci in anaerobic culture media. Pending the definitive culture result, empirical intravenous antibiotic treatment was started with 1 g of ceftriaxone every 24 h. The patient was admitted to the hospital for treatment.

**Figure 2:**
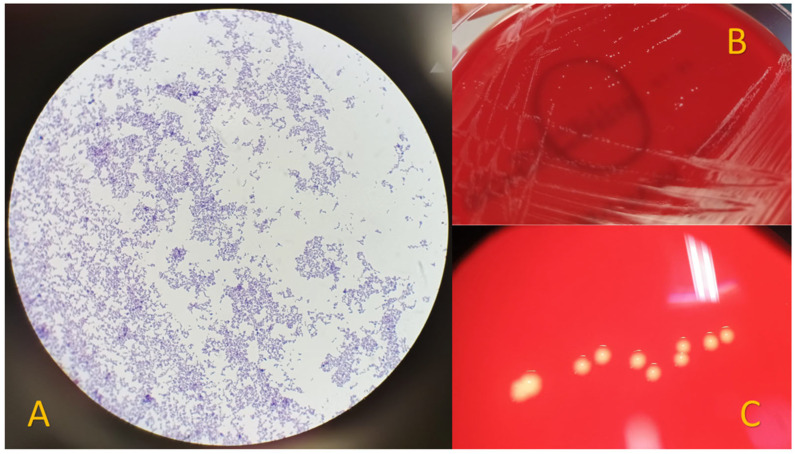
Isolation of *Parvimonas micra* from synovial fluid. (**A**) Gram stain showing Gram-positive cocci. (**B**) Visualization on Schaedler agar plates after 72 h of incubation at 37 °C under anaerobic conditions. (**C**) Visualization of smooth, small, white/yellowish colonies with a magnifying glass.

The clinical questioning was prolonged. The patient stated that she had no history of fever, constitutional or systemic symptoms, trauma or local lesions, animal contact, infections or dental procedures in the previous months. Laboratory blood tests showed elevated acute phase reactants (CRP 36 mg/L, ESR 57 mm/h) without leukocytosis, and uric acid levels of 4.2 mg/dL (historical levels always below 6). The study was completed with a transthoracic echocardiogram with no evidence of endocarditis and a chest X-ray with no abnormal findings. Blood cultures were sterile at 72 h, and other serologies were requested with a negative result. *Parvimonas micra* was isolated in a synovial fluid sample (Figure 2). While waiting for the antibiogram, the intravenous antibiotic treatment was changed to 1 g of ampicillin every 8 h (this treatment was maintained after confirming the correct and high sensitivity). Antibiotic susceptibility testing was performed using MIC gradient strips (BioMeriux, France) following the manufacturer’s instructions, resulting in penicillin 0.016 mg/L, imipenem 0.023 mg/L, clindamycin 0.016 mg/L, vancomycin 0.25 mg/L, and metronidazole 0.016 mg/L after 48 h of incubation at 37 °C in an anaerobic atmosphere. A third arthrocentesis was performed with extraction of 12 cc with the same polarized light microscopy characteristics as previously observed, and *Parvimonas micra* was isolated again.

During the first week of hospital stay, two additional arthrocenteses were necessary due to recurrence of knee inflammation despite the use of intravenous antibiotics. These synovial fluids were sterile, without isolation of *Parvimonas micra*. Due to this evolution, the dose of ampicillin was increased after 7 days to 1 g every 6 h after one week of treatment. Fourteen days after admission and 7 days after the antibiotic dose increase, no changes were observed, so the orthopaedic surgery department was contacted to perform joint lavage and debridement. Following this procedure, the knee showed great clinical and functional improvement. Twenty-three days after admission, the patient was discharged on oral ampicillin 1 g every 8 h until completing 8 weeks of treatment. Knee synovial surgery biopsy showed no acute inflammation or the presence of microorganisms via Gram and PAS stains, and the cultures were sterile.

Fifteen months after admission, the patient has had no further episodes of inflammation, maintained her baseline mechanical knee pain treated with analgesics, and was being followed up by the orthopaedic surgery department with an aim to assess a medium- to long-term prosthesis.

## 3. Discussion

Riesbeck et al. were the first authors to describe septic arthritis due to *Parvimonas micra* in a healthy native joint [9], and Stoll et al. described septic arthritis in a prosthetic joint [10]. This anaerobic Gram-positive coccus, named *Peptostreptococcus micros* or *Micromonas micros* until 2006, is part of the oral and gut microbiota and can appear as cocci in chains, aggregates, or in pairs (Figure 2). Synovial fluid from a septic joint infected by *Parvimonas micra* does not present typical septic arthritis characteristics, and systemic symptoms are rare, in contrast to other microorganisms. *Parvimonas micra* is a fastidious and slow-growing bacterium that needs enriched culture media and anaerobic atmospheres [11]. Therefore, if not suspected, its diagnosis can be easily missed and the infection therefore underdiagnosed. It is widely known that both delayed diagnosis and treatment of septic arthritis, regardless of the onset and virulence of the causative microorganism, can lead to joint destruction and loss of function. However, in recent years, advances in culture techniques and new methods for the identification of microorganisms, such as MALDI-TOF and 16 rRNA sequencing, have led to increased diagnosis of *Parvimonas micra*.

Few studies on *Parvimonas micra* septic arthritis have been published to date [2,9,11,12,13,14,15] and are summarized in Table 1. Of the eight patients previously described in the literature, seven (87.5%) were male, the same percentage of patients were over 60 years of age, and six (75%) had cases detected in a knee joint. Regarding comorbidities, four (50%) patients had a history of dental procedures or periodontal disease, two (25%) had a history of neoplasm, and one (12.5%) was under immunosuppressive treatment. Regarding the synovial fluid study, five (71.4%, calculated out of seven due to available data) had inflammatory features and four (50%) had chondrocalcinosis. One patient had a history of gout and was the only case in which *F. nucleatum* was isolated apart from *Parvimonas micra* [16]. This was the only case with an abscess and, consequently, features of septic fluid. Five patients (62.5%) required joint surgery in addition to antibiotics for complete recovery. After this literature review, our case appeared to be in line with the previous reported cases, as it occurred in an elderly patient with a history of neoplasm and a crystal arthropathy (mainly chondrocalcinosis), with involvement of a knee joint, with inflammatory joint fluid, and requiring surgery for full recovery. Although with this small number of published cases it is difficult to establish a pattern or risk factors relating *Parvimonas micra* septic arthritis, the reverberation of some clinical–epidemiological features seems more frequent and could help clinicians suspect it.

Sometimes behind persistent subacute or chronic arthritis, even with the presence of monosodium urate or calcium pyrophosphate crystals, the hidden cause is an infection by an anaerobic microorganism. Crystal arthropathies such as chondrocalcinosis [17] produce chronic inflammatory joint damage and an increased risk of septic arthritis. *Parvimonas micra* seems to be no exception, as reported in the literature (Table 1) and in our case. However, it is also important to note that chondrocalcinosis is more common in older patients and that the mean age of the patients was advanced, which is a possible bias.

Any state of immunosuppression or disruption of the immune system is a risk factor for developing septic arthritis [1,3], and tumors are an example. Riesbeck et al. (1999) and Ryan et al. (2021) [15] were the first ones to describe septic arthritis due to *Parvimonas micra* in patients with an oncological pathology, such as multiple myeloma or non-Hodgkin lymphoma. Subsequently, in 2017, Roy et al. [13] reported a renal and pancreatic transplant patient with septic arthritis due to *Parvimonas micra*, possibly related to its immunosuppression. Our presented case, although with a breast carcinoma in remission, could be in line with the tumor history of these cases.

Remote infections are widely known risk factors for septic arthritis, since the causative microorganisms can spread through the bloodstream. In the case of *Parvimonas micra*, although it is a common gut bacterium, none of the septic arthritis cases reported (Table 1) nor the one reported by us had a definite oral or digestive focus. However, a previous dental procedure seems to be a possible origin. This epidemiological background may be useful in the diagnostic suspicion.

Regarding treatment, most studies suggest that *Parvimonas micra* is a microorganism susceptible to multiple antibiotics used against anaerobes, such as β-lactams, clindamycin, and metronidazole, although isolates resistant to clindamycin and vancomycin have been found [18,19]. However, there is an important limitation with all studies of antimicrobial susceptibility testing (AST) with anaerobes due to different methodologies in AST techniques, lack of breakpoints or minimum inhibitory concentration (MIC) distributions to establish the categories “susceptible” (S) or “resistant” (R) in many species, such as *Parvimonas micra*, and different criteria between antibiogram committees. Our isolate presented low MICs for the tested antibiotics, with all of them below the MIC values that the EUCAST (European Committee on Antimicrobial Susceptibility Testing) considers should be cautiously encouraged. Empirical treatment is important due to the delay of isolation and AST results, and penicillins, carbapenems, clindamycin, and metronidazole are recommended. In targeted therapy, penicillins have been the most widely used targeted treatment when resistance is ruled out [1,18].

The novelty of this study is to highlight a rare pathology (eight cases described so far in the literature), most of the time neglected because it is underestimated and unknown. The take-home message is particularly addressed to rheumatologists and consists of taking this germ into account, especially in men over 60 years of age with a history of tumors and crystal arthropaties (mainly chondrocalcinosis) who have subacute arthritis, mainly involving the knee joint, with inflammatory synovial fluid and no response to conventional treatment. In these cases, thinking about *Parvimonas micra* and taking the appropriate cultures can clarify the diagnosis and prevent terrible outcomes with the correct treatment.

## 4. Conclusions

All types of septic arthritis should be suspected, evaluated, identified, and treated early to avoid fatal systemic and local consequences. Septic arthritis is a differential diagnosis that should always be included among the possible causes of arthritis in the day-to-day work of the rheumatologist, not only in an acute, but also in a subacute or chronic scenario, as there are pathogens with larval evolution and behavior that may go unnoticed, such as *Parvimonas micra*. There are few published data that can draw a specific spectrum of suspicion or risk factors for this difficult-to-grow, anaerobic, Gram-positive coccus, but in the cases described to date, *Parvimonas micra* seems to be more prevalent in older men and shows a preference for the knee joint, with inflammatory synovial fluid (not septic), a frequent history of tumor, possible concomitant crystal arthropaty (mainly chondrocalcinosis), and a frequent need for surgery for an adequate and complete recovery of the joint.

## Figures and Tables

**Table 1:** *Parvimonas micra* septic arthritis published to date. N, none; NR, not reported; M, male; F, female; CRP, C-reactive protein; ESR, erythrocyte sedimentation rate; CPPD, calcium pyrophosphate deposition disease. Age in years.

Author	Sex/Age	Oncological/ Immunosuppression History	Previous Dental Procedure or Disease	Affected Joint	Clinical Presentation	Synovial Fluid Characteristics	Associated CPPD	Blood Laboratory Findings	Treatment
Risbeck [9]	M/86	Multiple myeloma	N	Knee	NR	NR	Yes	NR	- Cloxacillin
Baghban [11]	M/65	N	Dental intervention two months before	Knee	NR	Inflammatory	Yes	- CRP 295 mg/dL	- Clindamycin - Arthrotomy + joint lavage
Dietvorst [12]	F/68	N	N	Knee	Acute	Bloody–inflammatory	Yes	- CRP 160 mg/dL - No leucocytosis	- Clindamycin
Roy [13]	M/61	Kidney and pancreatic transplant	Dental cleaning six months before	Knee	Chronic	Bloody–mechanical	No	- CRP 10 mg/dL	- Vancomycin + cefepime - Ceftriaxone + metronidazole - Arthrotomy + joint lavage
Sultan [2]	M/73	N	N	Knee	Acute	Inflammatory	Yes	- CRP 22 mg/dL - ESR 57 mm/h	- Vancomycin + penicillin - Arthrotomy + joint lavage + debridement
Ali [14]	M/77	N	Molar crown placement two days before	Knee	Subacute	Inflammatory	No	- CRP 119.4 mg/dL - ESR 48 mm/h - No leucocytosis	- Metronidazole - Debridement
Ryan [15]	M/65	Non-Hodgkin lymphoma	N	Hip	Subacute	Inflammatory	No	- CRP 29 mg/dL - ESR 89 mm/h	- Ampicillin sulbactam + doxycycline - Arthrotomy
Saishoji [16]	M/40	N	Severe periodontal disease	Sterno clavicular	Subacute	Abscess (purulent)	No	- CRP 1.9 mg/dL - ESR 38 mm/h	- Ampicillin sulbactam - Amoxicillin+ clavulanate

## Data Availability

Not applicable.

## References

[1] He M., Arthur Vithran D.T., Pan L., Zeng H., Yang G., Lu B., Zhang F. (2023). An update on recent progress of the epidemiology, etiology, diagnosis, and treatment of acute septic arthritis: A review. Front. Cell. Infect. Microbiol..

[2] Sultan A.A., Cantrell W.A., Khlopas A., Cole C., Piuzzi N.S., Sodhi N., Brooks P., Mont M.A. (2018). Acute septic arthritis of the knee: A rare case report of infection with *Parvimonas micra* after an intra-articular corticosteroid injection for osteoarthritis. Anaerobe.

[3] Mathews C.J., Weston V.C., Jones A., Field M., Coakley G. (2010). Bacterial septic arthritis in adults. Lancet.

[4] Piccinini D., Bernasconi E., Carelli M., Luvini G., Di Benedetto C., Lucchini G.M., Barda B., Bongiovanni M. (2023). *Parvimonas micra* a new potential pathogen in hospitalized patients: A case series from 2015–2022. Eur. J. Clin. Microbiol. Infect. Dis..

[5] Uemura H., Hayakawa K., Shimada K., Tojo M., Nagamatsu M., Miyoshi-Akiyama T., Tamura S., Mesaki K., Yamamoto K., Yanagawa Y. (2014). *Parvimonas micra* as a causative organism of spondylodiscitis: A report of two cases and a literature review. Int. J. Infect. Dis..

[6] Watanabe T., Hara Y., Yoshimi Y., Fujita Y., Yokoe M., Noguchi Y. (2020). Clinical characteristics of bloodstream infection by *Parvimonas micra*: Retrospective case series and literature review. BMC Infect. Dis..

[7] Cesta N., Foroghi Biland L., Neri B., Mossa M., Campogiani L., Caldara F., Zordan M., Petruzziello C., Monteleone G., Fontana C. (2021). Multiple hepatic and brain abscesses caused by *Parvimonas micra*: A case report and literature review. Anaerobe.

[8] Cobo F., Rodríguez-Granger J., Sampedro A., Aliaga-Martínez L., Navarro-Marí J.M. (2017). Pleural effusion due to *Parvimonas micra*. A case report and a literature review of 30 cases. Rev. Esp. Quimioter..

[9] Riesbeck K., Sanzén L. (1999). Destructive Knee Joint Infection Caused by *Peptostreptococcus micros*: Importance of Early Microbiological Diagnosis. J. Clin. Microbiol..

[10] Stoll T., Stucki G., Brühlmann P., Vogt M., Gschwend N., Michel B.A. (1996). Infection of a total knee joint prosthesis by peptostreptococcus micros and propionibacterium acnes in an elderly RA patient: Implant salvage with longterm antibiotics and needle aspiration/irrigation. Clin. Rheumatol..

[11] Baghban A., Gupta S. (2016). *Parvimonas micra*: A rare cause of native joint septic arthritis. Anaerobe.

[12] Dietvorst M., Roerdink R., Leenders A.C.A.P., Kiel M.A., Bom L.P.A. (2016). Acute Mono-Arthritis of the Knee: A Case Report of Infection with *Parvimonas micra* and Concomitant Pseudogout. J. Bone Jt. Infect..

[13] Roy M., Roy A.K., Ahmad S. (2017). Septic arthritis of knee joint due to *Parvimonas micra*. BMJ Case Rep..

[14] Ali H., Amir W., Bolick N.L. (2021). An uncommon case of native joint septic arthritis by *Parvimonas micra*. Anaerobe.

[15] Ryan P.M., Morrey B.F. (2021). *Parvimonas micra* causing native hip joint septic arthritis. Bayl. Univ. Med. Cent. Proc..

[16] Saishoji Y., Mori K., Izumi Y. (2023). Sternoclavicular Septic Arthritis Caused by *Parvimonas micra* and *Fusobacterium nucleatum* Infection with Intra-articular Corticosteroid Administration. Intern. Med..

[17] Schlee S., Bollheimer L.C., Bertsch T., Sieber C.C., Härle P. (2018). Crystal arthritides—Gout and calcium pyrophosphate arthritis: Part 2: Clinical features, diagnosis and differential diagnostics. Z. Gerontol. Geriatr..

[18] Sárvári K.P., Rácz N.B., Burián K. (2022). Epidemiology and antibiotic susceptibility in anaerobic bacteraemia: A 15-year retrospective study in South-Eastern Hungary. Infect. Dis..

[19] Guérin F., Dejoies L., Degand N., Guet-Revillet H., Janvier F., Corvec S., Barraud O., Guillard T., Walewski V., Gallois E. (2021). In Vitro Antimicrobial Susceptibility Profiles of Gram-Positive Anaerobic Cocci Responsible for Human Invasive Infections. Microorganisms.

